# Evolution of Exchangeable Copper and Relative Exchangeable Copper through the Course of Wilson's Disease in the Long Evans Cinnamon Rat

**DOI:** 10.1371/journal.pone.0082323

**Published:** 2013-12-17

**Authors:** Françoise Schmitt, Guillaume Podevin, Joël Poupon, Jérôme Roux, Pierre Legras, Jean-Marc Trocello, France Woimant, Olivier Laprévote, Tuan Huy NGuyen, Souleiman El Balkhi

**Affiliations:** 1 INSERM UMR1064, Jean Monnet Hospital, Nantes, France; 2 HIFIH - Pediatric Hepatogastroenterology Team, University Hospital of Angers, Angers, France; 3 Laboratory of Biological Toxicology, Lariboisière Hospital, APHP, Paris, France; 4 SCAHU, Animal Facility, Medicine University of Angers, Angers, France; 5 Department of Neurology, Lariboisière Hospital, APHP, Paris, France; 6 Centre national de Maladie Rare Wilson, Lariboisière Hospital, APHP, Paris, France; 7 Analytical and Experimental Toxicology (C-TAC), Faculty of Pharmacy, University Paris Descartes, Sorbonne Paris Cité, Paris, France; 8 INSERM U1144, Variability of the Response to Psychotropic Drugs, Paris Descartes University, Sorbonne Paris Cité, Faculty of Pharmacy, Paris, France; Queen Mary University of London, United Kingdom

## Abstract

**Background:**

Wilson's disease (WD) is an inherited disorder of copper metabolism leading to liver failure and/or neurological impairment. Its diagnosis often remains difficult even with genetic testing. Relative exchangeable copper (REC) has recently been described as a reliable serum diagnostic marker for WD.

**Methodology/Principal Findings:**

The aim of this study was to validate the use of REC in the Long Evans Cinnamon (LEC) rat, an animal model for WD, and to study its relevance under different conditions in comparison with conventional markers. Two groups of LEC rats and one group of Long-Evans (LE) rats were clinically and biologically monitored from 6 to 28 weeks of age. One group of LEC rats was given copper-free food. The other groups had normal food. Blood samples were collected each month and different serum markers for WD (namely ceruloplasmin oxidase activity, exchangeable copper (CuEXC), total serum copper and REC) and acute liver failure (serum transaminases and bilirubinemia) were tested. Every LEC rat under normal food developed acute liver failure (ALF), with 40% global mortality. Serum transaminases and bilirubinemia along with total serum copper and exchangeable copper levels increased with the onset of acute liver failure. A correlation was observed between CuEXC values and the severity of ALF. Cut-off values were different between young and adult rats and evolved because of age and/or liver failure. Only REC, with values >19%, was able to discriminate LEC groups from the LE control group at every time point in the study. REC sensitivity and specificity reached 100% in adults rats.

**Conclusions/Significance:**

REC appears to be independent of demographic or clinical data in LEC rats. It is a very simple and reliable blood test for the diagnosis of copper toxicosis owing to a lack of ATP7B function. CuEXC can be used as an accurate biomarker of copper overload.

## Introduction

Wilson's disease (WD) is a rare autosomal recessive disorder of copper metabolism due to loss of function mutations in the gene encoding ATP7B protein (GeneBank: U03464.1) [1]. This protein acts as a copper ATPase transporter, particularly in the liver where it ensures both excretion of copper from the hepatocytes into the bile and incorporation of copper into apoceruloplasmin. This yields to an efficient and stable protein: holoceruloplasmin (Cp) [2]. Defective ATP7B protein leads to progressive accumulation of copper in the liver and other tissues, resulting in hepatic and/or neurological impairment. Once diagnosed, the disease must be treated by lifelong use of copper chelating agents or by zinc salt therapy [3], and can even in some extreme cases require liver transplantation [2], [4]. Absence or delay of treatment can lead to irreversible sequelae and even death.

The diagnosis of WD is based on a combination of clinical and biological findings and can be confirmed by genetic analysis. Specialized laboratories can identify up to 97% of WD patients [5]. Determination of copper in liver biopsy remains a common practice to confirm WD in some countries. However, liver biopsy is invasive and genetic testing is costly and not available everywhere. In addition, classic biological tests lack specificity to identify some cases of WD patients, especially when extrahepatic signs are not developed (i.e. with no Kayser-Fleischer ring or MRI imaging abnormalities). On the other hand, there is a need to diagnose WD in presymptomatic patients in order to start their decoppering therapy before the onset of the disease. A more rapid and cost-effective test is therefore required.

Exchangeable copper (CuEXC) and its derived Relative Exchangeable Copper (REC, ratio CuEXC/total copper %) has recently been proposed as a new biomarker for diagnosing WD in humans [6]. In that study, REC provided 100% specificity and sensitivity. CuEXC corresponds to the labile fraction of copper bond mainly to albumin [7], [8], [9]. An increase of CuEXC superior to normal levels is thought to reflect a blood and tissue copper overload that occurs after saturation of hepatocytes and the spillage of Cp unbound copper into the blood owing to hepatic cytolysis. However, in that study [6], almost all of the patients already had already had neurological and/or hepatic manifestations related to WD and were compared with healthy subjects, meaning that the sickest of the sick were compared with the “healthiest” of the healthy. In addition, it is possible that the onset of disease manifestations (9 months-60 years) [10], [11] could vary according to the nature of the mutation, environmental conditions (such as daily copper intake) and general health status (not related to WD hepatic or renal failure). In order to confirm the validity of REC as a specific and sensitive non-invasive biomarker regardless of biological status and environmental conditions, we tested it in a WD animal model and followed it up throughout the progression of the disease.

Among WD animal models, one of the most studied is the Long-Evans Cinnamon (LEC) rat [12], a natural mutant strain of Long-Evans rat discovered in Japan in 1983 [13]. As in WD, LEC rats suffer from an inherited disorder of copper metabolism due to a loss of function mutation in the *ATP7B* gene [14], [15], but without any reported neurological impairment [12], [16]. Its hereditary hepatitis has been correlated with a 900 bp lack at the 3′ end of ATP7B gene [17]. The sensitivity of this animal model to dietary copper [18] and the natural tendency to develop acute liver failure (ALF) make it suitable to evaluate the sensitivity and the specificity of copper related parameters even in extreme conditions. A copper profile in LEC rats includes reduction of serum level of copper, reduction of ceruloplasmin and ceruloplasmin oxidase activity (COA) and copper accumulation in the liver. In the majority of studies, an elevation of bilirubinemia and liver transaminases starts between 10 to 14 weeks of age, corresponding to the first acute liver failure episode [19], [20], [21].

Hence, the first aim of this study was to assess the ability of the REC to discriminate LEC rats (with an ATP7B mutation) from Long-Evans (LE) control rats (without ATP7B mutation), whatever their copper intake regimens or the progression of the underlying liver failure. The second aim was to determine the accuracy of CuEXC as a biomarker able to reflect copper overload before and after the onset of liver failure.

## Materials and Methods

### 2.1 Ethical statement, animal care and experimental procedures

Long-Evans Cinnamon rats were first purchased from IAR (Institute for Animal Reproduction, Ibaraki, Japan) and then bred at the animal facilities of Angers, France. Long-Evans (LE) rats were directly purchased from Janvier, Inc. (Janvier S.A.S, Le Genest St-Isle, France). The animals were housed at the animal facilities of Angers University Medical School (SCAHU) and received human care according to the guidelines of the French Agriculture Ministry. Long-Evans Cinnamon and Long-Evans rats were 6 weeks old at the beginning of the study and there were both males and females. They were maintained in a 12-hour light cycle and fed ad libitum. Their food was composed of either Altromin® 1320 diet normally dosed in copper (13 ppm) or Altromin® C1041 food containing less than 1 ppm of copper (0.367 mg/kg). Both were provided by GENESTIL®, Royaucourt, France. This experiment was authorized by the “Pays de la Loire” ethical committee for animal experimentation (CEEA.2012.12). On weaning, the animals were all kept on normal food for one week for acclimatization and then were given either normal or C1041 food. The daily clinical monitoring and blood sampling began at week 6. Blood was drawn from the retro-orbital sinus every four weeks or in case of clinical signs of liver failure. This procedure was performed under isoflurane general anesthesia (3% v/v in air), without additional use of analgesics.

Altogether, 15 LEC rats received normal food (8 males, 7 females), 9 LEC rats (6 males, 3 females) had C1041 food and 6 Long-Evans rats (3 males, 3 females) served as the control group which was also given normal food. Hereafter, these three groups will be referred to as LEC, C1041 LEC and LE, respectively.

Careful clinical observation of the LEC rats resulted in the gradation of the symptoms developed during acute liver failure (ALF). These signs included extension of jaundice, behavior of the rat, and weight variations, in agreement with veterinarian pain scales [22]. It allowed us to establish a classification of hepatic failure in 4 grades of severity, going from no clinical disease to fulminant hepatitis (Table 1). To our knowledge, this has not been performed elsewhere. Rats scoring up to 6 were humanely sacrificed under general anesthesia by exsanguination, and blood samples as well as liver biopsies were processed.

**Table 1:** Acute liver failure intensity scale in the LEC rat.

Score	Mucocutaneous appearance	Behavior	Weight
**0**	Exclusively pink	Hyperactive	Gain
**1**	Yellow ears and/or tail	Quiet	Stable
**2**	Yellow ears, tail and snout	Response to stimuli	Loss
**3**	Yellow ears, tail, snout and feet	Dying	

The sum of the numbers obtained in each column attributes of a grade for the intensity of hepatic failure as follows:

0: no hepatic disease;

–3: mild hepatic failure; 1

–5: moderate hepatic failure; 4

–8: fulminant hepatitis. 6

### 2.2 Liver function tests

Serum total bilirubin and alanine (ALT) and aspartate (AST) aminotransferases were measured in the Department of Biochemistry at the Nantes University Hospital.

### 2.3 Serum ceruloplasmin oxidase activity

The enzymatic oxidase activity of ceruloplasmin is directly correlated to its functionality in the serum, meaning that copper has been properly incorporated in apoceruloplasmin by the action of the ATP7B protein in the liver. Hence, COA is a direct reflection of the functionality of ATP7B in hepatocytes and is collapsed in WD patients [23]. Ceruloplasmin oxidase activity was measured with *o*-dianisidine dihydrochloride (4, 4′-diamino-3,3′-dimethoxy-biphenyl) as substrate (Sigma-Aldrich France, Saint-Quentin Fallavier, France), as described by Schosinsky in 1974 [24]. Absorbance was measured at 540 nm on a Beckman-Coulter DU®640 spectrophotometer. Ceruloplasmin oxidase activity was defined as “625*absorbance” and reported in units/liter (U/l).

### 2.4 Serum Copper concentration, CuEXC and REC

The technical approach for the determination of exchangeable copper has already been published [6], [25]. Briefly, blood samples were collected and centrifuged at 3,000 rpm for 10 min. Serum was immediately frozen at −80°C, sent in dry ice to be analyzed in fewer than 7 days at the laboratory of Biological Toxicology at Lariboisière Hospital (Paris). Serum was thawed for 20 minutes at room temperature. It was then diluted with EDTA 3 g.L^−1^ (1∶1) and incubated for one hour at room temperature before ultrafiltration on Amicon® Ultra-4® filters with a 30 kDa cutoff (Millipore, Molsheim, France). The measurements of copper in ultrafiltrates were performed by Electrothermal Atomic Absorption Spectrometry (ETAAS) (5100, Perkin Elmer, Les Ulis France). Total serum copper was measured by the same instrument. REC was calculated following the formula: REC  =  CuEXC/Total serum copper %.

### 2.5 Statistical analysis

Statistical analysis was performed using R® 2.13.1 software, with a significance level at P<0.05. For quantitative values, Wilcoxon's paired test or Mann-Whitney test were used as comparison tests for small samples, and unpaired *t*-test for larger ones.

Received Operating Curves (ROC), sensitivity (SE) specificity (SP) and likelihood ratio analysis were established using GraphPad Prim® 5.04 for Windows (GraphPad Software, San Diego, CA), with confidence intervals (CI) fixed at 95%.

## Results

### 3.1 Liver disease evolution in the LEC rats

**Figure 1:**
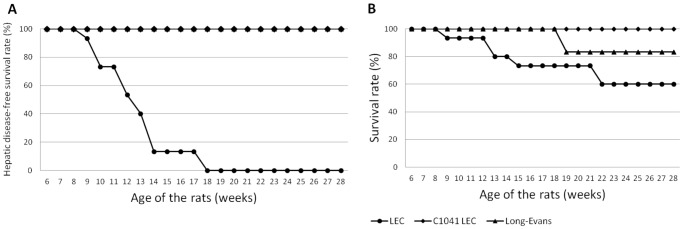
Survival and disease-free survival curves of the rats. Figure 1A shows acute liver failure present in every Long-Evans Cinnamon (LEC) rat under normal feeding, compared with C1041-fed LEC rats or to control Long-Evans (LE) rats. Figure 1B provides survival rates of the three groups of rats during the 28-week study.

All LEC rats under normal feeding presented clinical signs of ALF owing to their underlying pathology during the course of the experiment at a median age of 13 weeks (9–18). There was a broad range of severity in the development of ALF from moderate jaundice of ears and tails without behavioral changes to severe mucocutaneous jaundice with complete exhaustion of the animal leading to death within 24 hours. As expected, no rat in the C1041 LEC group or in the LE control group showed signs of ALF during the 28 weeks of monitoring (Figure 1a).

The death rate in the normally fed LEC rat group was as high as 40% at the end of the study. Five rats in this group spontaneously died of ALF (sex ratio: 1∶1), half of them with patent signs of ALF and one rat was euthanized with fulminant liver failure. The females tended to develop severe ALF sooner than the males with a median age of death at 13 weeks (9–21) versus 15 weeks (13–22), but this was not statistically significant. No rat in the C1041 LEC rat group died (Figure 1b), and one male in the LE control group was found dead at 18 weeks from what appeared to be a fight with its congeners.

#### Transaminases

**Figure 2:**
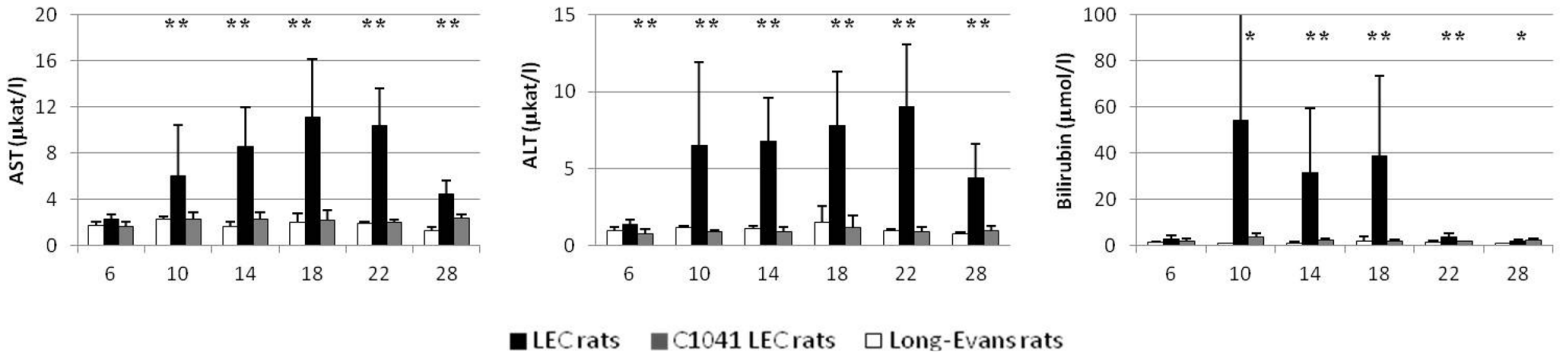
Evolution of transaminases over time in the different groups of rats. Alanine aminotransferase (ALT) and aspartate aminotransferase (AST) values are given in terms of means and standard deviation for each group at each time point, measured in weeks of life. One star (*) represents a statistically significant difference between the LEC and the LE group, and a double star (**), the difference between the LEC group and both the LE and C1041 LEC groups.

The usual ALF serum markers were evaluated in the LEC rats and their evolution during the course of the disease was monitored (Figure 2). Normal values were assessed in the LE control group as being inferior to 2.5 and 1.5 µkat/L for AST and ALT respectively. Serum transaminases revealed a pathological increase in the LEC group at 10 weeks of age, compared with C1041 LEC rats and control LE rats. Their values peaked at approximately 20 weeks (AST: 11.1 +/− 5.1 µkat/L and ALT: 9.0 +/− 4.1 µkat/L) before they began a progressive decrease but without reaching normal values again. In the C1041 LEC group, no increase in transaminases levels could be found. Total bilirubinemia showed the same evolution with an increase from the first clinical signs of ALF and with values correlated to the intensity of liver damage, reaching up to 500 µmol/L in case of fulminant liver failure. Contrary to the transaminases, bilirubinemia decreased to normal values after the end of an ALF episode.

### 3.2 Copper related markers

**Figure 3:**
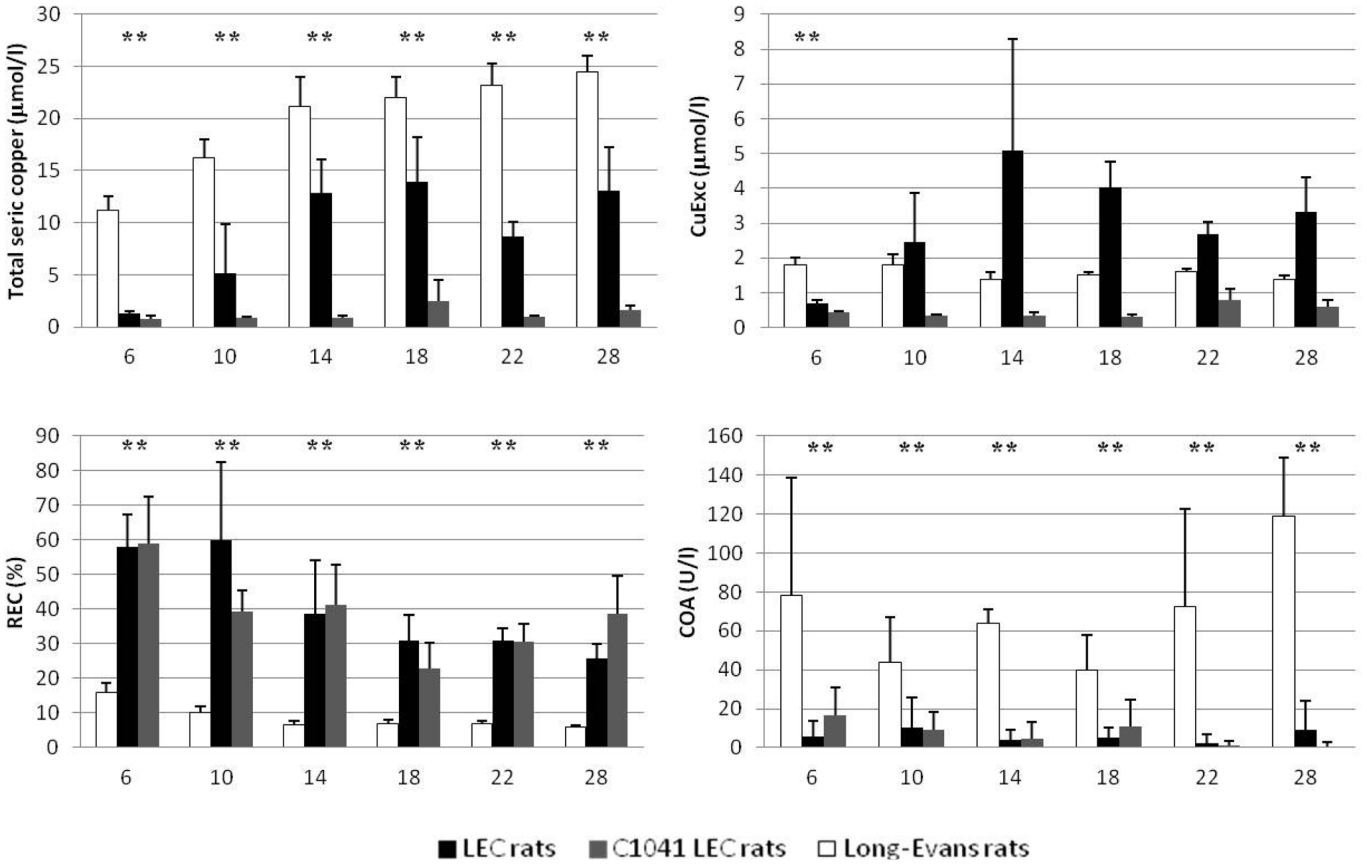
Evolution of Wilson's disease serum markers over time in the different groups of rats. Values are given in terms of means and standard deviation for each group at each time point, measured in weeks of life. The usual biological markers of WD are total serum copper and ceruloplasmin oxidase activity (COA), and new evaluated tools are represented by exchangeable copper (CuExc) and relative exchangeable copper (REC). A double star (**) represents the difference between the LE group and both the LEC and C1041 LEC groups.

Copper related biomarkers varied in an age-related manner (Figure 3). Indeed, at 6 weeks, total serum copper, CuEXC, and COA were significantly lower and REC was significantly higher in all of the LEC rats compared with the LE control group. These markers evolved during the rest of the study. We tested sensitivity (SE) and specificity (SP) for two distinct groups: young rats (6 to 10 weeks) and adult rats (14 to 28 weeks).

#### Total serum copper

Total serum copper in LE rats significantly increased over time (P<0.005) with a mean value at 6 weeks of 11.2 +/− 1.3 µmol/L versus 24.4 +/−1.6 µmol/L at week 28 (Figure 3). This increase became non-significant in the LE adult rats (>10 weeks old).

**Figure 4:**
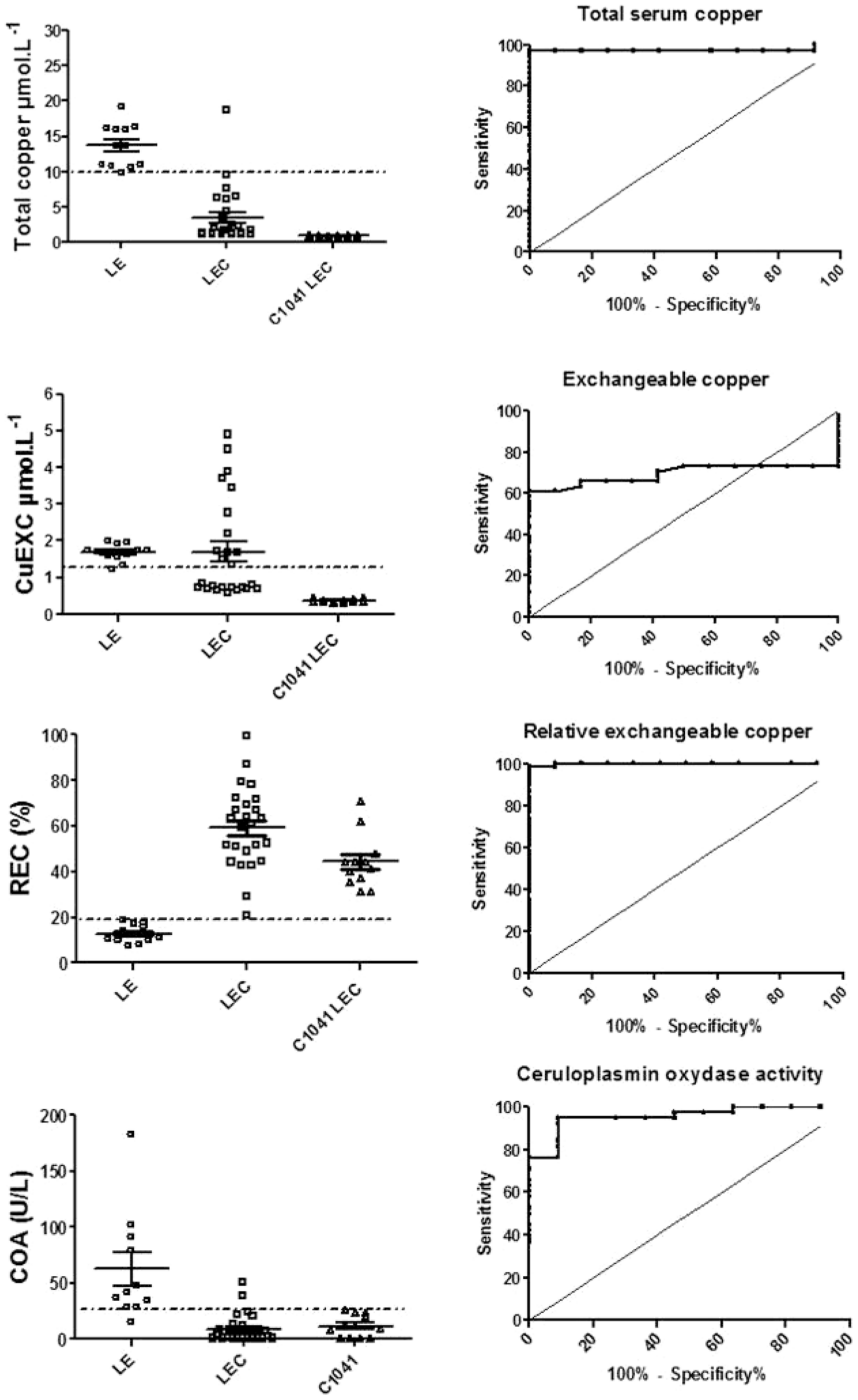
ROC tests for Wilson's disease biomarkers in young LEC, C1041 LEC and Long-Evans rats and associated LEC versus LE rat ROC curves. Total serum copper, exchangeable copper (CuExc), relative exchangeable copper (REC) and ceruloplasmin oxidase activity (COA) young rat results are shown. Broken lines represent the cut-off value obtained from ROC analysis.

**Figure 5:**
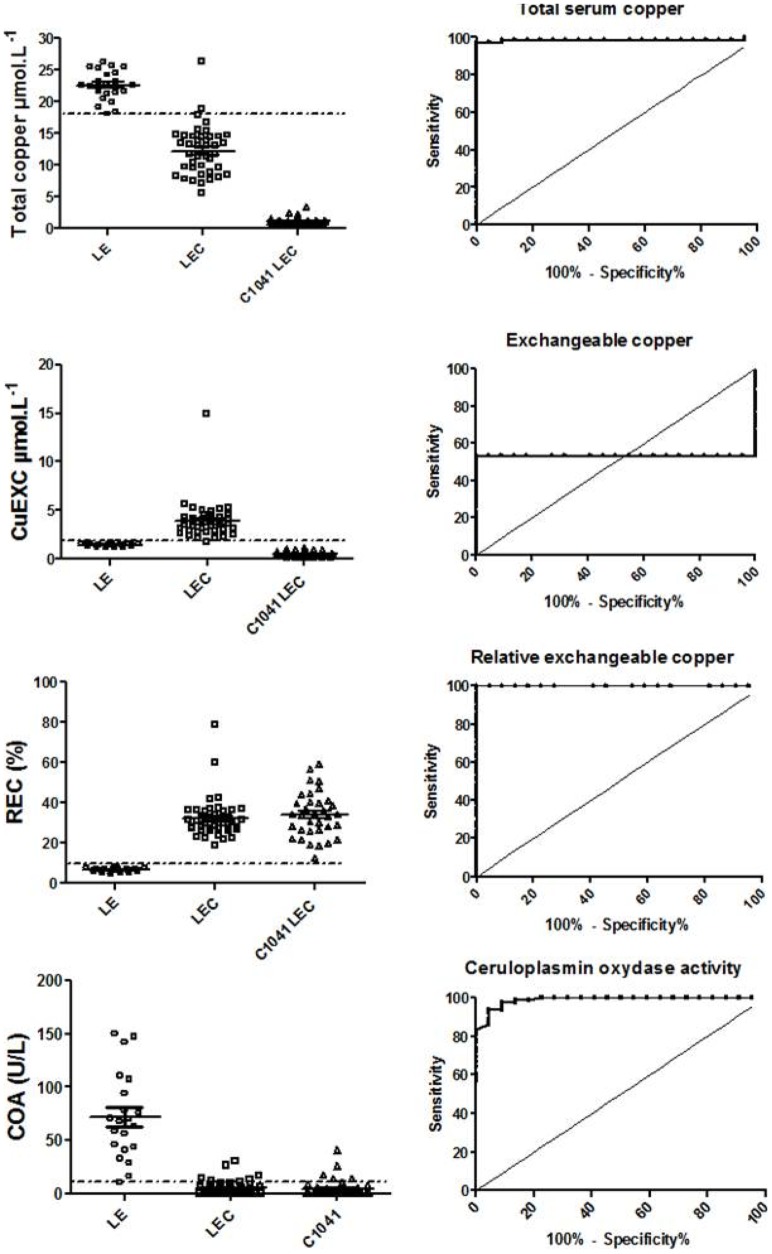
ROC tests for Wilson's disease biomarkers in adult LEC, C1041 LEC and Long-Evans rats and associated LEC versus LE rat ROC curves. Total serum copper, exchangeable copper (CuExc), relative exchangeable copper (REC) and ceruloplasmin oxidase activity (COA) in adult rat results are shown. Broken lines represent the cut-off value obtained from ROC analysis.

In young rats, total serum copper cutoff at 10.2 µmol/L resulted in an SE 97.3% and SP 91.7% (Figure 4 and Table S2). In the adult rats (>10 weeks old), a cutoff at 18.0 µmol/L yielded a SE 97% and SP 98% (Figure 5 and Table S2).

**Figure 6:**
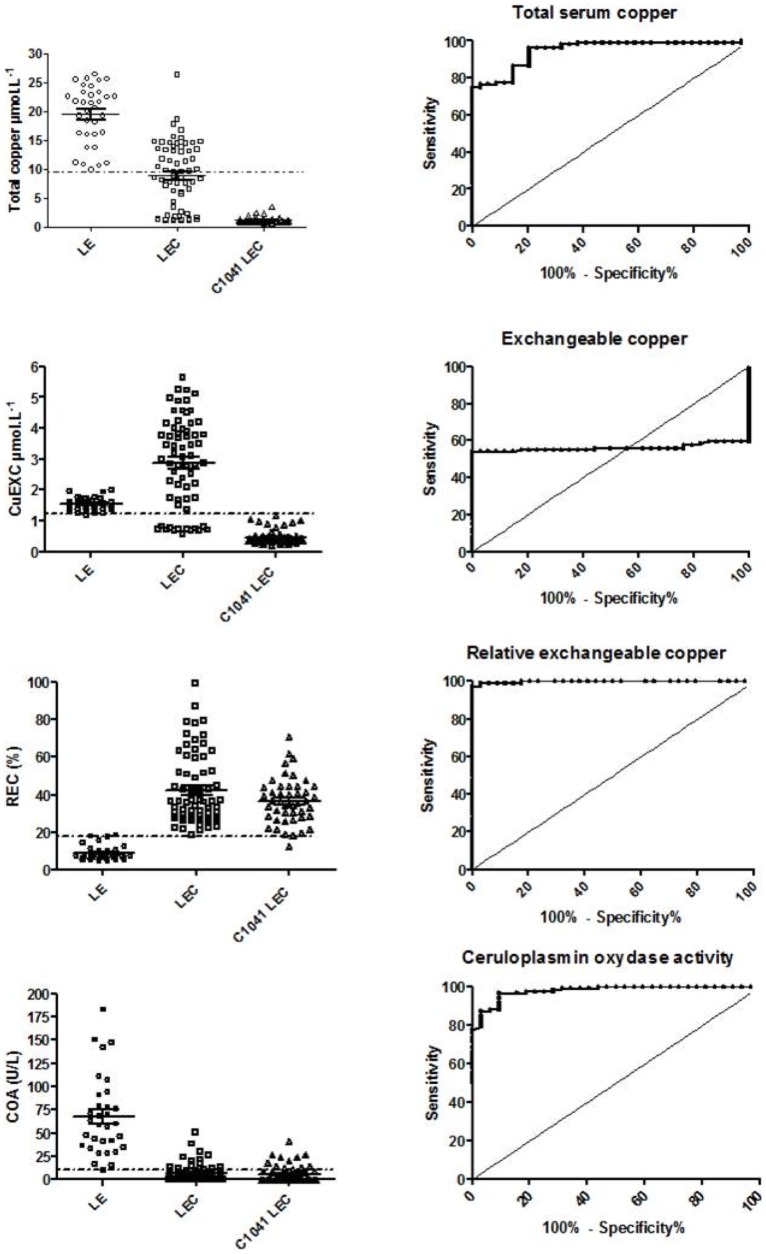
Global ROC tests for Wilson's disease biomarkers in LEC, C1041 LEC and Long-Evans rats and associated LEC versus LE rat ROC curves. Total serum copper, exchangeable copper (CuExc), relative exchangeable copper (REC) and ceruloplasmin oxidase activity (COA) in adult rats results are shown. Broken lines represent the cut-off value obtained from ROC analysis.

With the values taken together (regardless of age, liver failure and copper regimen), a cutoff set at 10.5 µmol/L yielded poor sensitivity (76.6%) but good specificity (97.1%) (Figure 6 and Table S1). Liver failure in LEC rats on normal food caused a significant increase in serum copper levels which then partially overlapped with LE rat values.

#### Exchangeable copper

Long-Evans CuEXC values remained relatively constant over time and always inferior to 2.0 µmol/L, whereas LEC CuEXC values varied with liver failure, becoming significantly higher than LE values after 10 weeks (P<0.001) (Figure 3). C1041 LEC values remained systematically lower than 1.15 µmol/L throughout the study, i.e. lower than LEC and LE levels. The mean CuEXC LEC rat value at 6 weeks was lower than that of the LE control rats (0.71 +/− 0.07 µmol/l vs. 1.8 +/− 0.2 µmol/l, P = 0.001) but like total serum copper, CuEXC values were also dependent on copper intakes and ALF. Thus, CuEXC was unable to discriminate all of the LEC from the LE control group and yielded very low sensitivity and specificity (Figures 4 and 5).

#### Relative exchangeable copper

**Figure 7:**
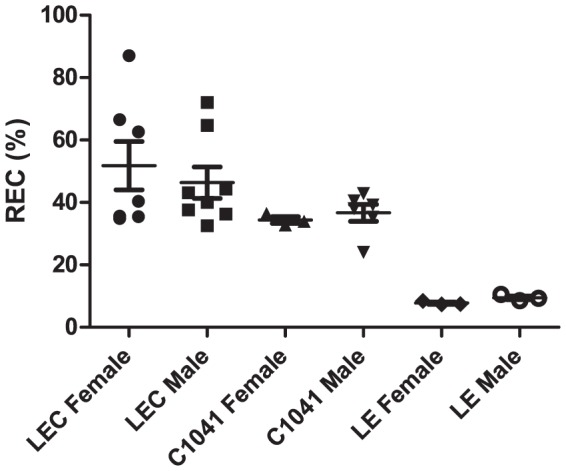
Influence of gender on REC values. Mean +/− SEM values are given separately for females and males in each group of LEC or LE rats.

**Figure 8:**
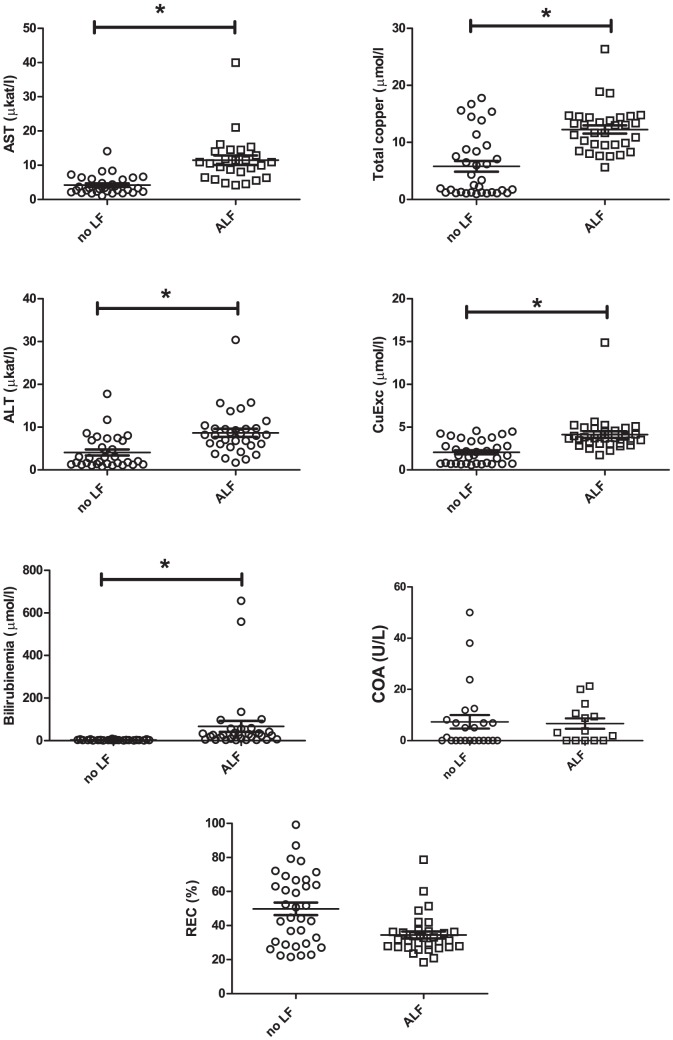
Comparison of serum biomarkers according to the presence or absence of acute liver failure. Values, means and SEM are given. One star (*) represents a statistically significant difference between the two groups.

In both the LEC and C1041 LEC groups, REC was significantly higher than in the LE control group (P<0.01) at every time point of the study. Mean values were at 49.5% +/− 16.6% in the LEC group, 36.5% +/− 3.8% in the C1041 LEC group, versus 8.9% +/− 1.2% in the LE control group. A cutoff set at 18.2% offered SE and SP at 98% and 100%, respectively, in young rats. A cutoff set at 10.5% offered 100% SE and 100% SP for adult rats (Figures 4 and 5, Table S2). Taking the young and adult values together, a cutoff set at 19.0% enabled the marker to discriminate both LEC groups from the LE control group with 97.3% sensitivity and 100% specificity (Figure 6 and Table S1). REC values were not significantly different between male and female rats for all groups (Figure 7). In addition, REC was independent of weight and remained discriminating whatever the copper regimens (Figures 4 and 5) and the degree of liver failure, as shown below (Figure 8).

#### Ceruloplasmin oxidase activity

Ceruloplasmin oxidase activity revealed the same type of results with LE mean values (68.7 +/− 16.6 U/l) always superior to LEC (6.3 +/− 3.7 U/l) and C1041 LEC (6.0 +/− 3.9) values (P<0.01) (Figure 3). Nevertheless, great inter-individual and intra-individual disparities were observed and were greater with the COA assay than with other copper related markers. In the young rats, a cutoff at 27.2 U/L yielded 94.7% SE and 90.9% SP. In the adults, a COA cutoff at 16.6 U/L provided SE 93.7% and SP 95.5% (Figure 5, Table S2).

#### Copper related markers evolution in acute liver failure

There were great inter-individual disparities at each time point owing to the different ages at the onset of ALF. We therefore studied the evolution of different markers before and during ALF (Figure 8). For this analysis, we only used the values of the LEC rat group.

AST and ALT were 2- to 3-fold higher than normal values in cases of clinical ALF (11.47 +/− 7.11 µkat/l vs. 4.18 +/− 2.75 µkat/l. P<0.0001 and 8.66 +/− 5.34 µkat/l vs. 4.08 +/− 3.90 µkat/l. P<0.0001. respectively). Mean bilirubinemia ranged from 2.88 +/− 1.97 µmol/l to 66.88 +/− 145.8 µmmol/l. The total serum copper of LEC rats after the onset of ALF (12.3 +/− 4.1 µmmol/l) approached levels equivalent to the values of the LE control group (19.3 +/− 1.5 µmol/l). The mean value of CuEXC increased from 2.05 +/− 1.36 µmol/L before the onset of ALF to 4.13 +/− 2.18 µmol/L during ALF (Figure 8).

There was a slight but significant decrease in REC values after ALF (34.5 +/− 11.9% vs. 49.8% +/− 11.2% before ALF, P = 0.004), but values during ALF did not reach the cutoff of 19%. COA did not show any significant difference before or after the onset of ALF (Figure 8).

**Figure 9:**
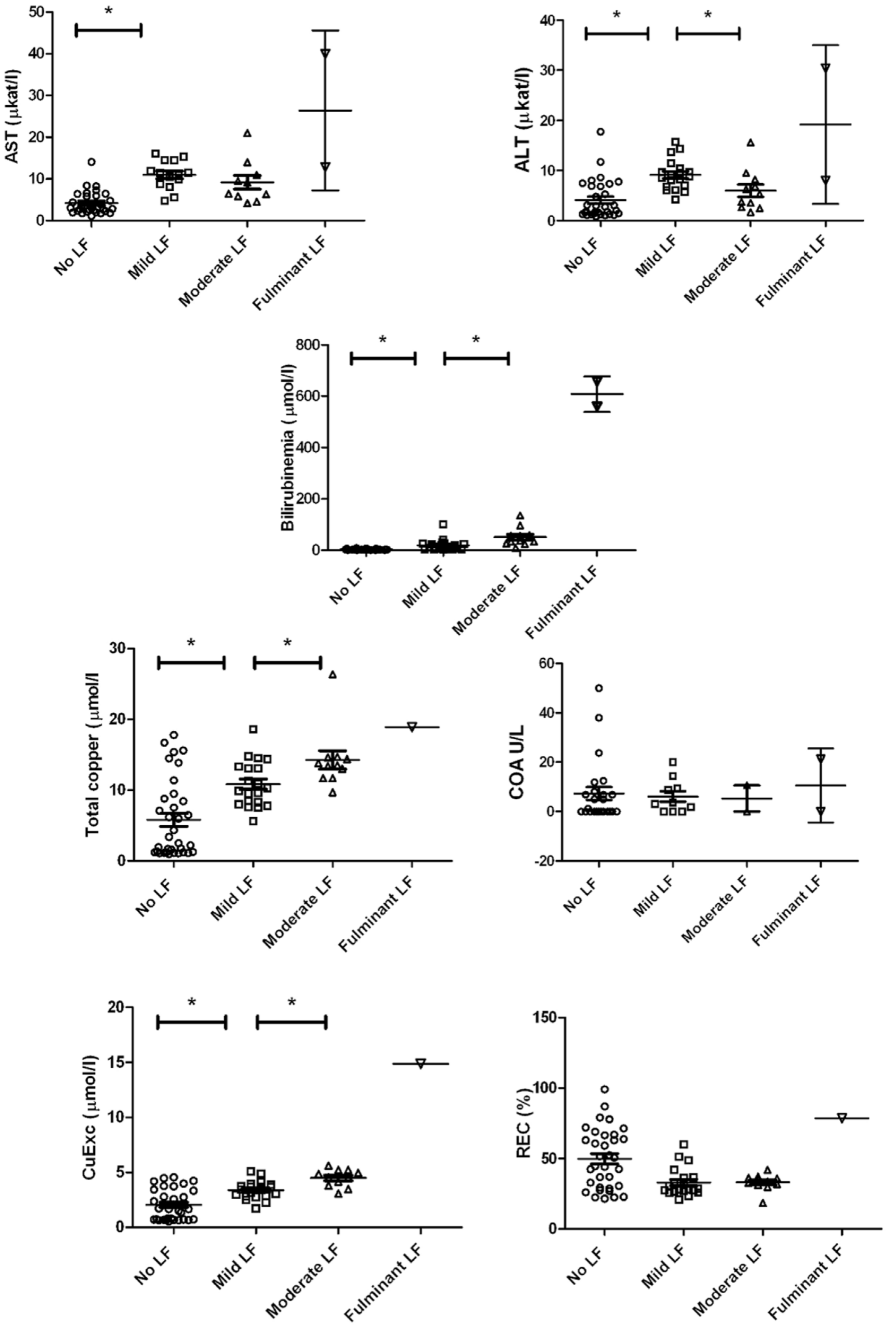
Evolution of serum biomarkers according to the grade of severity of acute hepatic failure. Values, means and SEM are given. One star (*) represents a statistically significant increase in serum marker value between the two grades of ALF.

We then attempted to correlate the values of these markers with the degree of ALF as defined in Table 1 (Figure 9). Despite the small number of rats in the moderate and fulminate liver failure groups (scoring 4–5 and 6–8 according to Table 1), we observed a correlation between the degree of liver failure and bilirubinemia, total serum copper and CuEXC, respectively. COA and REC were not influenced by the degree of liver failure, confirming their ability to discriminate LEC regardless of hepatic dysfunction.

## Discussion

### 4.1 REC as a WD diagnostic tool in LEC rats

In a previous study in humans [6], REC appeared to be an excellent marker for the diagnosis of WD, with 100% SP and SE among the tested subjects. Nevertheless, this first study only dealt with patients with suspected WD and REC served as discriminatory factor between the WD patients and the healthy subjects, regardless of sex, age or the degree of underlying liver failure. We therefore sought to test the validity of this new biomarker throughout the course of the liver disease, from a presymptomatic stage to the beginning of chronic liver failure, and to study the potential effects of copper depletion on the evolution of REC values. Owing to the sensitivity of Long Evans Cinnamon rats to dietary copper and their tendency to develop liver failure, this WD animal model enabled us to test REC even in extreme conditions.

In this study, we demonstrated that REC could be used as a non-invasive and reliable diagnostic tool for WD in LEC rats. REC remained discriminating between LEC and LE rats throughout the study and was, in particular, not influenced by the presence of liver damage or by the copper intake regimen. Despite the fact that REC tended to decrease in adult rats and during ALF, LEC values always remained superior to 19%, with excellent sensitivity and specificity. Taking only adult rat values into account, the sensitivity and specificity of REC was 100%. Nevertheless, the decrease in REC values between 6 to 14-week-old Long-Evans rats and their stability after the age of 14 weeks indicated that it could be necessary to establish specific standards in a young population. In addition, it is well established that copper metabolism varies according to age [13].

Giving copper-free food to LEC rats prevented them from developing any clinical or biological signs of ALF throughout the course of this experiment. This was in agreement with other results [18]. Although these rats did not show disease-related symptoms, REC was elevated in all rats from the beginning of the study. On the contrary, total serum copper and exchangeable copper varied according to the copper intake regimen and the degree of liver failure. Total serum copper yielded good SE and SP in young and adult rats (one at a time) but the number of false positives was too high after the onset of ALF.

Clinical observation of our LEC rats was in agreement with data in the literature [13], [15], [19], [27] with at least one episode of ALF before the age of 20 weeks in 100% of the cases without specific treatment. Among them, about 40% died of this first acute episode of liver failure and the rest survived with poor clinical and biological signs of chronic liver disease such as chronic jaundice and mild elevation of ALT and AST. In this group, transminases were within normal ranges until the age of 10 weeks and bilirubinemia levels were normal after ALF had stopped. On the contrary, total serum copper increased during ALF to the values found in the LE control group. The only markers that could discriminate LEC rats throughout the study were COA (in agreement with Merle et al. [23]) and REC. However, COA has been reported to be almost absent in the LEC strain in some studies [26] but has been detected in others [19]. In our LEC rats, COA had great inter and intra-individual disparities.

Further studies are required in order to guarantee the specificity of REC in differentiating WD from other kinds of liver disease. Preliminary results using a model of cholestasis in rats showed that REC values were considerably lower than REC values in our animal model (data not shown).

### 4.2 CuEXC evolution during liver failure

Within the population of LEC rats, our results indicate that total serum copper and CuEXC levels were correlated to ALF. CuEXC in LEC rats started to increase with elevation of the hepatic cytolysis markers (ALT and AST) and its values remained high as the ALF settled.

As suggested in previous studies [18], [28], [29], copper is supposed to progressively accumulate in LEC hepatocytes during the first weeks of life until cytosolic storage capacities are overwhelmed. This could be accelerated by a massive burden of dietary copper as recently reported by Siaj et al. [18]. Liver accumulated copper induces liver deterioration through oxidative stress and cell cycle activation and is finally released in the blood – not bound to ceruloplasmin – then eliminated through kidney ultrafiltration. This leads to an elevation of urinary copper. CuEXC has been described as the labile fraction of copper bound to different molecules such as albumin and transcuprein [8]. Hence, an increase in CuEXC serum levels could indicate the presence of abnormally high amount of toxic extrahepatic copper.

Another potential use of CuEXC could be as a marker of decoppering therapy efficiency. In fact, we have observed that CuEXC levels did not vary with time in the group of C1041 LEC rats whose copper intakes were quasi-absent and always remained under 1.0 µmol/l. Zinc-salt therapy, for instance, acts by inducing metallothionein synthesis in the enterocytes [30], which sequestrate copper and eliminate it through feces [31]. Overtime, it depletes copper from the organism by a negative imbalance between intakes and outtakes. It is for this reason that CuEXC could be a good and simple marker for long-term monitoring with a persistent low value being the sign of good observance. Further studies will therefore be needed to assess the potential use of CuEXC as a monitoring marker in rats under chelating or zinc salt therapy.

Since CuEXC corresponds to the labile fraction of copper not bound to ceruloplasmin, the only factor that could a priori cause REC values to evolve toward normal values should be the restoration of holoceruloplasmin levels in the serum which is controlled by a functional ATP7B protein in the liver [32], [33]. This hypothesis must be validated in a WD patient with a liver transplantation and could have a future application in pre-clinical trials in LEC rats by helping the following up of WD gene therapy.

## Conclusion

In summary, our results indicate that REC could be used as a reliable WD diagnostic marker with 100% sensitivity and 100% specificity. REC is at least as good as COA to discriminate LEC (ATP7B-) from LE (ATP7B+) rats. In addition, it does not depend on the existence of underlying liver damage or on the copper intake regimen. On the other hand, we propose the use of exchangeable copper as a marker which able to reflect copper overload before and during ALF. Finally, in comparison with normal Long-Evans rats, we can propose cut-off values for each serum marker tested in this WD animal model.

## Supporting Information

Table S1 **Results of ROC curve analysis of serum markers for Wilson's disease.**
(DOC)

Table S2 **Detailed ROC curve analysis of Wilson's disease markers in young and adult LEC rats.**
(DOC)
