# Supplementary material for: Evolution of Exchangeable Copper and Relative Exchangeable Copper through the Course of Wilson's Disease in the Long Evans Cinnamon Rat
Source: PLoS One. 2013 Dec 17;8(12):e82323. doi: 10.1371/journal.pone.0082323 (PMC3866119; doi:10.1371/journal.pone.0082323)
Supplement: Table S1 — Results of ROC curve analysis of serum markers for Wilson's disease. (DOC) [file pone.0082323.s001.doc]

**Table S1: Results of ROC curve analysis of serum markers for Wilson's disease.**

| Biological marker | Cutoff values for WD diagnosis | SE  (%) | SP  (%) | PLR | NLR | AUC  (95% CI) |
| --- | --- | --- | --- | --- | --- | --- |
| Ceruleoplasmin oxidase activity (COA) | < 27.2 U/l | 96.6 | 90.6 | 10.3 | 0.04 | 0.979  (0.959-0.998) |
| Total serum copper | < 10.5 µmol/l | 76.6 | 97.1 | 2.6 | 0.24 | 0.947  (0.911-0.983) |
| Relative exchangeable copper (REC) | > 19.0% | 97.3 | 100 | / | 0.03 | 0.998  (0.994-1.002) |

Cutoff values are given for each test, as well as sensitivity (SE), specificity (SP), positive likelihood ratio (PLR), negative likelihood ratio (NLR) and area under the curve (AUC) with its 95% confidence interval (95% CI).
