# Supplementary material for: Evolution of Exchangeable Copper and Relative Exchangeable Copper through the Course of Wilson's Disease in the Long Evans Cinnamon Rat
Source: PLoS One. 2013 Dec 17;8(12):e82323. doi: 10.1371/journal.pone.0082323 (PMC3866119; doi:10.1371/journal.pone.0082323)
Supplement: Table S2 — Detailed ROC curve analysis of Wilson's disease markers in young and adult LEC rats. (DOC) [file pone.0082323.s002.doc]

**Table S2:** detailed ROC curve analysis of Wilson's disease markers in young and adult LEC rats

|  | **Young (6-10 weeks-old) LEC rats** | | | | **Adults (>10 weeks-old) LEC rats** | | | |
| --- | --- | --- | --- | --- | --- | --- | --- | --- |
| Biological maker | Cutoff value for WD diagnosis | SE (%) | SP (%) | AUC  (95% CI) | Cutoff value for WD diagnosis | SE (%) | SP (%) | AUC  (95% CI) |
| Ceruleoplasmin oxydase activity (COA) | < 27.2 U/l | 94.7 | 90.9 | 0.955  (0.898-1.012) | <16.6 | 93.7 | 95.5 | 0.988  (0.972-1.005) |
| Total serum copper | <10.2 µmol/l | 97.3 | 91.7 | 0.975  (0.927-1.024) | <18 µmol/l | 97.0 | 98.0 | 0.986  (0.961-1.011) |
| Relative exchangeable copper (REC) | >18.2% | 98.0 | 100 | 0.999  (0.994-1.003) | >10.5% | 100 | 100 | 1.00  (1.00-1.00) |

Cutoff values are given for each test, as well as sensitivity (SE), specificity (SP) and area under the curve (AUC) with its 95% confidence interval (95% CI).
